# Identifying the Determinants of Egg Food Quality, and the Structural Relationship Between Egg Food Quality, Trust, and Loyalty: The Case of the U.S. Market

**DOI:** 10.3390/nu18030452

**Published:** 2026-01-29

**Authors:** Myungkeun Song, Joonho Moon, Luo Jing

**Affiliations:** 1 Department of Tourism Management, Dong-A University, Gudeok-ro, Seo-gu, Busan 49236, Republic of Korea; 2 Department of Tourism Administration, Kangwon National University, Chuncheon 24341, Republic of Korea; 3 College of Business, Tourism Management Department, Xi’an International University, Xi’an 710021, China

**Keywords:** egg, trust, loyalty, price fairness, nutritional value, product size, packaging, hygiene

## Abstract

Background/Objectives: Eggs serve as an important source of nutrition for the general public. However, despite their importance, research examining consumer characteristics related to egg consumption remains limited, focusing on the quality. Therefore, the primary objective of this work is to define the concept of food quality from the consumer’s perspective in the U.S. egg market. This work employs five attributes to evaluate the food quality of eggs, including price fairness, nutritional value, product size, packaging, and hygiene. Methods: This research also investigates the structural relationships between food quality attributes, trust, and loyalty. Food quality is operationalized through five sub-dimensions: price fairness, nutritional value, product size, packaging, and hygiene. Data were collected via an online survey using the Clickworker platform, resulting in 311 valid responses for statistical analysis. Covariance-based structural equation modeling was employed to test the proposed hypotheses. Results: The findings reveal that trust is positively influenced by price fairness, nutritional value, packaging, and hygiene. Furthermore, loyalty is positively affected by nutritional value, product size, and trust. Conclusion: This research contributes to the literature by offering a consumer-centered definition of egg quality and by identifying key quality-related attributes that influence trust and loyalty.

## 1. Introduction

According to Peri [1], food quality is a multifaceted construct that can be conceptualized in diverse ways depending on the specific food product under consideration. This perspective underscores that consumers do not rely on a single, uniform standard when evaluating quality; rather, they apply differentiated evaluative criteria that are contingent upon the intrinsic and extrinsic characteristics of each food type. Such heterogeneity in quality perception suggests that the meaning of “quality” is socially and contextually constructed through consumers’ experiences, expectations, and usage situations. Eggs, in particular, occupy a central position in everyday diets worldwide due to their relatively low price, ease of preparation, and high nutritional value, including their rich protein content and essential micronutrients [2,3,4]. Because eggs are frequently purchased and consumed across diverse demographic groups [3,4], consumers are likely to form well-developed yet potentially varied criteria for assessing their quality, such as freshness, shell appearance, size, safety, and perceived naturalness. Despite the dietary and economic significance of eggs, empirical research that explicitly examines egg quality from a consumer-oriented perspective remains limited. Much of the previous literature has tended to focus on technical, production-oriented, or nutritional assessments of egg quality [3,4,5,6], thereby overlooking how consumers themselves define and interpret high-quality eggs in everyday purchasing contexts. To address this gap in the literature, the present study aims to systematically investigate consumer perceptions of egg quality and to identify the key attributes that shape these perceptions. In doing so, this study seeks to contribute to a more nuanced understanding of food quality by incorporating the consumer’s viewpoint into the discussion of egg quality evaluation.

This research attempts to conceptualize egg quality by delineating a set of relevant sub-dimensions. First, eggs are valued for providing high-quality nutrients at a relatively low cost, which underscores the importance of price fairness [2,3,4,5]. Additionally, egg size is likely to become a salient attribute for consumers, as it is associated with the intake of an appropriate quantity of food [6,7]. Scholars emphasized that packaging is important in terms of protecting the product [8,9], while hygiene is considered crucial due to its close association with consumer health [10,11]. Therefore, packaging and hygiene could become critical, given that eggs are highly perishable and susceptible to breakage; thus, these attributes play an essential role in ensuring product safety and consumer satisfaction.

Next, the current research incorporates the constructs of trust and loyalty as key attributes in understanding consumer behavior. Trust represents a belief in the reliability and integrity of a product, and has been extensively examined in prior literature as a pivotal factor influencing consumer evaluations and decision-making processes [12,13]. Similarly, loyalty, which is closely linked to sustained purchasing behavior and increased sales, has been widely studied in the field of consumer behavior [14,15]. These constructs are expected to serve as effective mechanisms for evaluating whether the proposed framework adequately explains behavioral responses to perceived egg quality.

All things considered, the first objective of this work is to conceptualize egg food quality through five key dimensions: price fairness, nutritional value, product size, packaging, and hygiene. Building upon these dimensions, the study further seeks to explore the structural relationships among perceived egg quality, consumer trust, and loyalty. The findings are anticipated to contribute meaningfully to the literature by elucidating consumer-driven determinants of food quality. Moreover, the study holds theoretical significance by exploring the extent to which food quality attributes can account for the formation of trust and loyalty toward eggs. From a practical perspective, the results may provide actionable insights to producers, retailers, and supply chain stakeholders by delivering consumer-centered information regarding quality perceptions in the egg market.

## 2. Literature Review and Hypotheses Development

### 2.1. Food Quality of the Egg

Scholars argued that food quality was defined differently depending on the type of food product under consideration [1,16]. This variation occurred because the factors consumers considered when evaluating and purchasing food products varied across product categories [1,17]. Accordingly, prior research attempted to define food quality attributes specific to different food domains. For instance, Halimi et al. [18] investigated food quality attributes in the context of halal food, Tian et al. [19] examined the quality dimensions of tea products, and Lee et al. [20] explored food quality in the coffee market using a multidimensional approach. In addition, Indiarto et al. [21] inspected the characteristics of meat quality, while Manisha and Jagadeeshwar [22] identified the main attributes defining food quality in dairy products. Collectively, these studies noted that food quality had been widely researched across diverse food categories, underscoring the importance of a product-specific approach to food quality conceptualization.

In the context of egg consumption, price emerged as a particularly salient attribute. Consumers generally made purchasing decisions based on price, and eggs, as essential and frequently purchased goods, exhibited relatively high price sensitivity as a sort of low involvement food [2,3]. In this regard, scholars defined price fairness as the extent to which consumers perceived a given price as reasonable or justifiable, and it served as a critical indicator in consumer price evaluations [23,24,25]. Prior studies claimed that perceptions of unfair pricing reduced perceived value and negatively influenced purchase decisions, even for staple food products such as eggs [2,23].

Beyond price considerations, the nutritional value of eggs could be highlighted as one of their primary advantages. Eggs were recognized for their rich nutritional profile and their important role in promoting individual health because it contains adequate amount of protein and fat [2,4]. Supporting this view, Rhaman et al. [5] demonstrated that nutritionally rich foods, such as organic products, contributed to improved health conditions in the minds of consumers. As consumer interest in health promotion increased, nutritional value became an essential determinant of consumer behavior in food markets, according to researchers [26,27,28]. Indeed, empirical evidence documented that consumers’ food decision-making was significantly influenced by perceptions of nutritional value [29,30].

Next, product size was identified as an imperative food quality attribute, as it reflected the amount of food available for consumption and influenced consumers’ perceptions of value relative to cost [31,32]. In the case of eggs, researchers reported that egg size significantly affected consumers’ evaluations and preferences [6,7,33]. Hieke et al. [32] confirmed that portion size is an essential attribute to explain individual decision-making of food consumption.

Packaging was likewise considered a critical attribute, as it helped preserve food safety and facilitated handling and consumption, thereby shaping consumer perceptions of product quality [8,9,34]. The extant literature documented that consumer perceptions of food products were substantially influenced by packaging attributes, which are related to the marketing communication and customer experience [8,35]. Researchers alleged that food packaging functioned as an important factor because it provided information about the product, protected food from external shocks and harmful substances, and created a first impression for consumers [36,37].

Finally, hygiene—referring to the cleanliness and sanitary conditions under which food was produced, handled, and distributed—played a crucial role in shaping consumer perceptions of food quality [38,39]. Food produced under unsanitary conditions posed potential health risks, making hygiene an essential evaluative criterion for consumers [11,38]. Flanagan and Soon-Sinclair [39] found that consumers preferred food providers and establishments where hygiene standards were properly maintained because food-related illnesses can arise from food that is handled and managed under unsanitary conditions. Previous works posited that food hygiene played a crucial role in reducing the consumption of unsafe food and mitigating adverse health effects perceived by consumers [40,41]. Based on the review of the literature, this study proposed five key attributes of food quality in the context of egg consumption: price fairness, nutritional value, product size, packaging, and hygiene.

### 2.2. Trust

Trust is a form of belief that consumers hold toward a particular product [42,43]. Because consumers tend to continue purchasing products they trust, prior studies have actively explored the concept of trust [12,13]. Nelson and Kim [43] adopted trust to investigate the characteristics of news subscribers. Zhang et al. [44] chose trust as a main attribute to scrutinize the behavior of mobile shopping service users. Yang et al. [12] examined celebrity chef food consumers using trust as a focal attribute. The literature review noted that trust has been studied in various consumer research sectors by numerous scholars, meaning that it could become an essential piece to figure out consumer behavior.

### 2.3. Loyalty

Loyalty refers to the degree to which consumers are willing to consistently use a particular product [43,44]. Consumers who exhibit loyalty play a crucial role in increasing the sales of specific companies or brands, which is why scholars have continuously researched this topic [14,15]. Malik et al. [13] adopted loyalty to explore the behavior of library service users. Yum and Kim [45] also inspected the determinants of loyalty, focusing on entertainment platform users. Su et al. [14] investigated the influential attributes of loyalty in the case of a food delivery application system. Attar et al. [46] employed loyalty as a dependent variable to understand the behavior of online food shoppers. Integrating the literature review, it can be inferred that loyalty has been widely investigated as a dependent variable by numerous researchers.

### 2.4. Hypotheses Development

Temperini et al. [47] and Skripnuk et al. [48] contended that food quality is an imperative attribute to build consumer trust. Taylor et al. [49] also claimed that consumer trust in the fruits and vegetables is significantly influenced by food quality. Moon et al. [50] implemented structural equation model analysis, and the findings demonstrated a positive association between trust and the food quality of coffee. It can be inferred that food quality is likely to build trust, and such a framework could be applied in the case of eggs. Therefore, this work proposes the following research hypotheses:

**Hypothesis 1a (H1a):**  *Price fairness positively affects the trust of the egg.*

**Hypothesis 2a (H2a):**  *Nutritional value positively affects the trust of the egg.*

**Hypothesis 3a (H3a):**  *Product size positively affects the trust of the egg.*

**Hypothesis 4a (H4a):**  *Packaging positively affects the trust of the egg.*

**Hypothesis 5a (H5a):**  *Hygiene positively affects the trust of the egg.*

Bihamtaet al. [51] and Majid et al. [52] researched hotel restaurant customers, and the findings uncovered a positive impact of food quality on loyalty. Zhong and Moon [53] demonstrated a positive relationship between food quality and loyalty by employing fast-food restaurant customers. Suhartanto et al. [54] investigated halal food consumers, and the findings revealed that loyalty is positively affected by food quality. Cui et al. [55] also noted that food quality perception is influential to the positive appraisal of the fresh food in the online commerce domain. Severt et al. [24] found that price fairness positively impacted loyalty among customers of local food restaurants. Yoo et al. [29] also disclosed a positive effect of nutritional value on loyalty to food stores. Sun and Moon [56] revealed a positive impact of portion size on customer loyalty to the beef products. Waheed et al. [36] uncovered that food packaging played an important role in building loyalty. Al-Zyoud et al. [38] investigated restaurant customers, and the results indicated that customer loyalty is positively influenced by hygiene. Integrating the review of literature, this work proposes the following hypotheses:

**Hypothesis 1b (H1b):**  *Price fairness positively affects loyalty to the egg.*

**Hypothesis 2b (H2b):**  *Nutritional value positively affects loyalty to the egg.*

**Hypothesis 3b (H3b):**  *Product size positively affects loyalty to the egg.*

**Hypothesis 4b (H4b):**  *Packaging positively affects loyalty to the egg.*

**Hypothesis 5b (H5b):**  *Hygiene positively affects loyalty to the egg.*

Sann et al. [15] found that loyalty is positively influenced by trust in the case of bus service. Attar et al. [46] and Cui et al. [55] reported a positive association between trust and loyalty in the online food market domain. Su et al. [14] also uncovered the positive impact of trust on loyalty by examining the food delivery application system users. In sum, numerous works exhibited that trust is likely to becomes an essential determinant of loyalty of consumers. Hence, the following research hypothesis is proposed:

**Hypothesis 6 (H6):**  *Trust positively affects loyalty to the egg.*

## 3. Method

### 3.1. Research Model and Description of the Measurement Items

**Figure 1:**
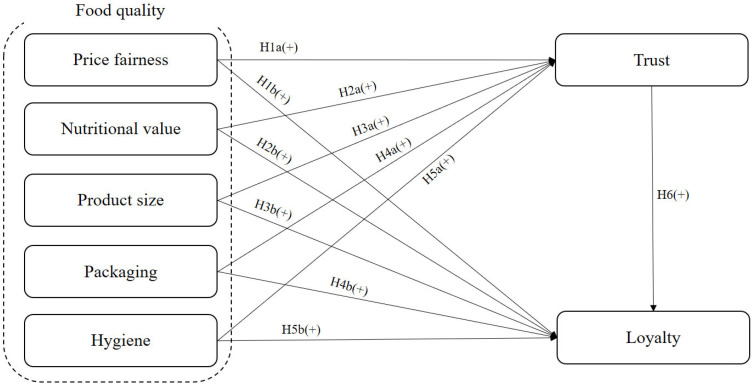
Research model.

Figure 1 is the research model. Food quality encompasses factors such as price fairness, nutritional value, product size, packaging, and hygiene. The attributes of food quality positively affect both trust and loyalty. Loyalty is also positively influenced by trust.

Table 1 presents the measurement items. This work adopted Likert 5-point scales (1 = strongly disagree, 5 = strongly agree) for the measurement. Measurement items were derived from the prior works, and the items were adjusted to be more adequate for the aim of the current work. Only hygiene was measured using three sub-items, and most constructs consisted of four measurement items. Most of the measurement items were derived from consumer-related studies published in journals indexed in the Scopus database. The studies used for item development were selected from the literature published within the past ten years. Based on the extracted items, this research synthesized measurement items from multiple prior studies to better align with the objectives of the present study. The following is the operational definition of the constructs:

Price fairness: How consumers appraise the price of an egg rationally.

Nutritional value: How eggs are useful for better health conditions.

Product size: The perceived consumption amount of the egg.

Packaging: How consumers assess the packaging quality of the egg.

Hygiene: The perceived status of cleanliness of the egg to be consumed.

Trust: The degree of confidence the consumer has in the reliability of the egg product.

Loyalty: Consumers’ intention to sustain the purchasing of eggs from the same suppliers.

### 3.2. Data Collection and Analysis

This study collected data using Clickworker (https://clickworker.com), a widely utilized crowdsourcing platform in quantitative social science research, and the use of this platform in prior works supports the credibility of the collected data and its suitability for statistical inference [56,57,58]. Also, this research adopted an online survey as the data collection method, considering that it allows respondents to participate in the survey in a comfortable setting, regardless of time or location. Data collection was conducted between 11 April and 13 April 2025, following the official announcement of the U.S. tariff policy. U.S. consumers were selected as survey participants because they were directly affected by the newly implemented tariff policy on egg imports during the data collection period. Accordingly, nationality was used as a screening criterion for participant eligibility. To maintain the objectivity of the findings, no additional screening criteria were applied, as eggs are a widely purchased and consumed product across demographic groups. Data were collected using convenience sampling through an online panel on the Clickwork platform, which was chosen for its accessibility and efficiency in reaching U.S. consumers. Respondents were instructed to answer based on their egg purchasing behavior within two weeks. A total of 311 valid responses were collected. This sample size is sufficient for the general guideline for statistical analysis, which recommends at least ten responses per measurement item to ensure the reliability of statistical inferences by the recommendation of the previous literature [59].

Table 2 provides the demographic profile of the respondents. The sample included 93 males and 218 females. Participants in their 30s and 40s comprised approximately 71.5% of the total sample. In terms of monthly household income, about 63% of respondents reported earning less than $5000. Educational attainment was distributed as follows: less than a college degree (n = 138), a bachelor’s degree (n = 115), and a graduate degree or higher (n = 58). Weekly egg consumption was reported as follows: less than once (n = 40), 2–4 times (n = 182), 5–8 times (n = 68), and more than 9 times (n = 21).

This study first conducted a frequency analysis to describe the demographic characteristics of the survey respondents. To assess convergent validity, confirmatory factor analysis (CFA) was employed. Following established guidelines, convergent validity was evaluated using multiple criteria: standardized factor loadings exceeding 0.50, average variance extracted (AVE) greater than 0.50, and construct reliability (CR) above 0.70 [59,60]. Subsequently, the means and standard deviations for each latent construct were calculated. Discriminant validity was evaluated using the Fornell–Larcker criterion, which requires that the square root of the AVE for each construct be greater than the corresponding inter-construct correlation coefficients [59,61]. This work additionally computed both the mean and standard deviation (SD) of the constructs.

To assess the goodness-of-fit of the measurement model, several fit indices were examined. These included a χ^2^/*df* ratio less than 3, a Root Mean Square Residual (RMR) below 0.05, and additional indices such as the Goodness-of-Fit Index (GFI), Normed Fit Index (NFI), Relative Fit Index (RFI), Incremental Fit Index (IFI), Tucker–Lewis Index (TLI), and Comparative Fit Index (CFI), all of which are considered acceptable at thresholds above 0.80. The Root Mean Square Error of Approximation (RMSEA) was also assessed, with values below 0.10 indicating an acceptable level of model fit [59,60,61]. Finally, covariance-based structural equation modeling was conducted to test the proposed research hypotheses at the 95% confidence level. For the statistical analysis, this work adopted both statistical packages in social science (SPSS) 18.0 version, and analysis of moment structure (AMOS) 21.0 version.

## 4. Results of the Empirical Analysis

### 4.1. Convergent Validity and Discriminant Validity of the Measurement Items

Table 3 illustrates the results of confirmatory factor analysis. The goodness of fit indices could become the evidence that the results are statistically acceptable (χ^2^ = 589.622, *df* = 303, χ^2^/*df* = 1.946, RMR =0.047, NFI = 0.930, RFI = 0.919, IFI = 0.957, TLI = 0.959, CFI = 0.965, RMSEA = 0.055). All factor loading, AVE, and CR values meet the criteria for the validity and reliability of the measurement items. Moreover, Table 3 describes the mean and SD ((price fairness (mean = 2.53, SD = 1.24), nutritional value (mean = 4.29, SD = 0.76), product size (mean = 4.17, SD = 0.84), packaging (mean = 4.21, SD = 0.83), hygiene (mean = 3.97, SD = 0.88), trust (mean = 3.97, SD = 0.89), and loyalty (mean = 3.92, SD = 0.97)).

Table 4 is the correlation matrix. All diagonal values (square root of AVE) are greater than off-diagonal values. It indicates that the discriminant validity is likely to be ensured. Moreover, loyalty positively correlated with trust (r = 0.516, *p* < 0.05), price fairness (r = 0.189, *p* < 0.05), nutritional value (r = 0.413, *p* < 0.05), product size (r = 0.488, *p* < 0.05), packaging (r = 0.467, *p* < 0.05), and hygiene (r = 0.440, *p* < 0.05). Also, trust positively correlated with price fairness (r = 0.240, *p* < 0.05), nutritional value (r = 0.460, *p* < 0.05), product size (r = 0.517, *p* < 0.05), packaging (r = 0.574, *p* < 0.05), and hygiene (r = 0.579, *p* < 0.05).

### 4.2. Results of the Structural Equation Model

Table 5 depicts the results of the structural equation model for hypothesis testing. The results revealed that trust is positively affected by price fairness (β = 0.094, *p* < 0.05), nutritional value (β = 0.207, *p* < 0.05), packaging (β = 0.256, *p* < 0.05), and hygiene (β = 0.257, *p* < 0.05). Loyalty is positively influenced by nutritional value (β = 0.207, *p* < 0.05), product size (β = 0.188, *p* < 0.05), and trust (β = 0.277, *p* < 0.05). All things considered, H1a (price fairness), H2a (nutritional value), H4a (packaging), and H5a (hygiene) were supported for trust, while H2b (nutritional value), H3b (product size), and H6 (trust) were supported for loyalty.

## 5. Discussion

### 5.1. Discussion of the Empirical Results

This research aimed to explore consumer characteristics in the U.S. egg market using data collected through an online survey. By focusing on eggs as a specific case of food quality evaluation, the study examined consumer behavior by incorporating trust and loyalty as key outcome variables. Drawing on Peri [1], this research conceptualized egg food quality as a multidimensional construct consisting of five sub-dimensions: price fairness, nutritional value, product size, packaging, and hygiene. By integrating these five quality-related factors into the analytical framework, the work demonstrated that they collectively served as effective and meaningful indicators for defining egg quality from the consumer’s perspective. The descriptive results indicated notable differences across the perceived quality attributes. In particular, price fairness received the lowest mean score among the measured dimensions (mean = 2.53), meaning that consumers experienced a relatively high level of uncertainty or concern regarding egg pricing. This perception was likely to be influenced by the timing of data collection, which coincided with disruptions in egg supply caused by external factors such as avian influenza. Such supply-side shocks might heighten price volatility and, in turn, weaken consumers’ perceptions of price fairness. In contrast, nutritional value recorded the highest mean score (mean = 4.29), indicating that consumers strongly recognized eggs as an essential food item with substantial health and nutritional benefits. This finding reflected the well-established perception of eggs as a nutrient-dense and affordable source of protein in everyday diets.

The results of the hypothesis testing further revealed differentiated effects of food quality attributes on trust and loyalty. Price fairness was found to significantly influence consumer trust, but it did not exert a direct effect on loyalty. This result suggested that while fair pricing perception contributed to the formation of trust, it alone was insufficient to sustain long-term consumer commitment or repeated purchasing behavior. Also, eggs could be a relatively inexpensive food ingredient as a low-involvement good that consumers commonly use to obtain essential nutrients at an affordable price, which may serve as a plausible explanation for the limited influence of price on egg-related evaluations. This aspect may be more pronounced in the U.S. market, as eggs are priced relatively lower than beef, which is a primary source of protein for many Americans, making eggs a more cost-efficient protein option. Additionally, the habitual nature of egg purchasing and consumption might be able to contribute to the formation of trust with respect to price; however, the findings suggest that this habitual behavior may have limitations in directly fostering consumer loyalty. In contrast, nutritional value significantly influenced both trust and loyalty, underscoring the central role of health-related considerations in shaping consumer evaluations and loyalty in the egg market. Consumers who perceived eggs as nutritionally valuable were more likely to trust the product and remain loyal over time. Namely, the findings indicate that consumers developed trust in the nutritional value provided by eggs, and this trust functions as a key motivation for continued consumption as a source of healthy protein. Next, product size was shown to have a significant effect on loyalty, indicating that the provision of appropriately sized eggs played an important role in encouraging repeat purchases. This finding implied that tangible product attributes, such as size, functioned as practical cues that directly affected consumers’ consumption experiences and value perceptions. Regarding trust, the non-significant effect of egg size may be explained by the possibility that larger eggs lead consumers to harbor doubts related to genetic modification or the use of chemical additives, thereby weakening any positive influence of size on trust. Consumers’ suspicions that egg size may have been manipulated through genetic modification or chemical agricultural practices could lead to concerns about potential negative health effects associated with egg consumption. Although egg size is an important attribute, eggs are also consumed for health-promoting purposes. Therefore, perceptions related to egg size may have had limited influence on consumer trust, as health considerations may outweigh size-related factors. Furthermore, the results indicated that packaging and hygiene significantly contributed to the formation of trust, as these attributes likely signaled safety, care, and quality assurance. However, neither packaging nor hygiene was found to significantly influence loyalty. It can be inferred that their effects may have been limited to initial evaluations rather than sustained consumer engagement. Next, egg packaging played a significant role in enabling consumers to purchase eggs with credibility, as it typically provides information about the supplier’s production methods and the manufacturing date. However, the appropriateness of packaging revealed a limited effect on consumer loyalty, as consumers tend to perceive packaging as a basic and expected component necessary for safe product storage for preventing egg breakage rather than as a differentiating factor that fosters loyalty. Similarly, in terms of hygiene, consumers generally infer the sanitary condition of eggs based on their external appearance, while having limited access to detailed information regarding the actual production and distribution processes. As a result, hygiene-related attributes may contribute to the formation of trust but have a limited role in generating consumer loyalty, which can be considered a plausible explanation for the study’s results. Finally, consumer trust was identified as a critical determinant of loyalty, noting its possible central mediating role in the relationship between food quality attributes and consumer loyalty. It can be inferred that trust functioned as a key psychological mechanism through which perceptions of food quality were translated into long-term loyalty. Overall, the results emphasized that while multiple food quality attributes shaped consumers’ credibility to the egg, only certain attributes—particularly nutritional value and product size—directly fostered loyalty, whereas others, including price fairness, packaging, and hygiene, primarily operated through the development of trust.

Regarding the results, this research could present a policy design. From a policy perspective, the results indicated that price fluctuations in essential food markets such as eggs influenced consumer trust. Measures focused on monitoring price changes and improving price transparency during periods of supply disruption might help stabilize consumer expectations. The findings also indicated that providing clear information about the nutritional value of eggs helped consumers understand food quality better through government regulation. Standardized labeling and public information initiatives might be able to play a role in facilitating informed decision-making. In addition, the results underscored the relevance of product standards related to size classification and labeling. Clear guidelines reduced ambiguity and supported consistent quality perceptions among consumers through government regulation. The importance of packaging and hygiene underscored the role of food safety regulations. Maintaining hygiene standards and encouraging compliance across the supply chain could contribute to consumer confidence in food safety in the process of food policy design. Lastly, the findings implied that policy initiatives encouraging responsible production and distribution practices supported trust in the egg market. Such efforts complemented existing regulatory frameworks without imposing excessive burdens on market participants.

### 5.2. Limitations and Suggestions for Future Research

This research has several limitations that could be acknowledged. First, the research focused exclusively on consumers in the U.S. Given that eggs are a widely consumed food product across diverse cultural and regional contexts, future works would benefit from examining consumer behavior in other countries to enhance the generalizability of the findings. Cross-cultural comparisons could provide a more comprehensive understanding of how perceptions of egg quality vary globally. Additionally, this study has limitations in representing the overall U.S. market, as the proportion of male respondents is relatively lower than that of female respondents, and participants aged 60 and older account for less than 3% of the sample. Given that the U.S. population has an approximately balanced sex ratio (about 50% male and 50% female) and that roughly 70% of the population falls within the 18–64 age range, the sample may not fully represent the demographic structure of the overall U.S. population [62]. Future research might be able to take this issue into account and seek to present findings based on samples that are more balanced in terms of demographic characteristics. In addition, the exclusive focus on eggs could be regarded as a limitation of this research. Building on this limitation, future research might be able to extend the scope to a wider range of food ingredients to provide further insights into consumers’ perceptions of food quality. Moreover, this research derived sub-dimensions through a review of the literature to define the food quality of eggs. Future research could identify a broader range of sub-dimensions related to food quality in the context of egg consumption by incorporating expert consultations or qualitative research approaches, which could include consumer knowledge, ethical concerns (e.g., animal welfare), or brand reputation. Next, the current work relied solely on a self-reported survey method, which might be subject to biases such as social desirability or limited introspective accuracy. Future research might be able to consider employing a broader range of methods—such as observational studies, experimental designs, or mixed-method approaches—to gain deeper insights into consumer behavior and decision-making processes related to egg consumption, considering longitudinal studies to assess loyalty over time.

## 6. Conclusions

### 6.1. Theoretical Implications

This study offers several theoretical contributions. First, this work identified five key dimensions—price fairness, nutritional value, product size, packaging, and hygiene—as critical elements in defining egg quality from the consumer’s perspective. By conceptualizing egg quality through these dimensions, the work might be able to provide a clearer understanding of how consumers perceive and evaluate quality in the egg market. Second, the research empirically demonstrated that these five quality attributes significantly accounted for the formation of trust and loyalty, thereby emphasizing their essential role in shaping consumer behavior in the context of staple food products such as eggs.

### 6.2. Managerial Implications

From the perspective of industry practitioners, the findings indicated that price stability was associated with the development of consumer trust. Given the routine consumption of eggs, relatively consistent pricing—particularly during periods of supply uncertainty such as avian influenza outbreaks—appeared to reduce consumer concerns related to price fairness. The results also disclosed that communicating the nutritional value of eggs was relevant for shaping consumer trust and repeat purchasing behavior. Marketing messages that emphasized health-related benefits were particularly relevant for consumers who placed importance on nutritional considerations. From a nutritional perspective, rather than emphasizing marketing messages that portray eggs simply as a healthy source of protein, it could be more effective to adopt marketing strategies that emphasize efforts to minimize potential concerns, such as exposure to endocrine-disrupting substances or stress experienced by hens during the rearing process. Additionally, product size was found to be related to consumer loyalty. This finding implied that managing consistency in product size, potentially through coordination with suppliers, contributed to more favorable consumer evaluations. In this context, supplier relationships represented one aspect of quality management in the egg market. Alternatively, egg-producing companies may consider investing in agricultural technologies that enable farms to produce larger eggs, thereby securing more competitive products. Packaging and hygiene were also associated with trust formation. Investing in packaging technology that adequately protects the product and conveys cleanliness supports positive quality perceptions. Similarly, maintaining acceptable hygiene standards throughout storage and display processes could reinforce perceptions of safety. Ultimately, the findings uncovered that transparent business practices and socially responsible activities could be linked to higher levels of consumer trust and loyalty. Practices such as ethical sourcing or environmentally conscious production might be able to contribute to a more positive overall brand image, which are associated with the trust and loyalty of consumers.

## Figures and Tables

**Table 1:** Description of the measurement items.

Construct	Code	Item	Reference
Price fairness	PF1 PF2 PF3 PF4	Egg prices were fair. Egg prices were reasonable. Egg prices were appropriate. Egg prices were acceptable.	Samoggia et al. [23] Severt et al. [24]
Nutritional value	NV1 NV2 NV3 NV4	Eggs promote health. Eggs contain many good nutrients. Eggs were essential for health promotion. Eggs provide good nutrients.	Yoo et al. [29] Hati et al. [30]
Product size	SZ1 SZ2 SZ3 SZ4	The size of the eggs was appropriate. The size of the eggs was suitable for consumption. The egg size was reasonable for consumption. The egg size was adequate for consumption.	Sun & Moon [56]
Packaging	PK1 PK2 PK3 PK4	The hygiene condition of the eggs was appropriate. The packaging condition of the eggs was good. The packaging of the eggs was appropriate. The egg packaging materials were used properly.	Waheed et al. [36] d’Astous & Labrecque [37]
Hygiene	HY1 HY2 HY3	Eggs were hygienic. Eggs were clean. Eggs were well-managed in terms of cleanliness.	Zhang et al. [40] Francioni et al. [41]
Trust	TR1 TR2 TR3 TR4	Egg is reliable. Egg is trustworthy. I have a trust to the egg. Egg is credible.	Yang et al. [12] Zhang et al. [44]
Loyalty	LY1 LY2 LY3 LY4	I am going to use the same eggs again. I will purchase the same eggs. I am willing to pay for the same eggs. I am willing to buy the same eggs.	Su et al. [14] Attar et al. [46]

**Table 2:** Information of survey participants (N =311).

Item	Frequency	Percentage
Male	93	29.9
Female	218	70.1
20s	40	12.9
30s	119	38.3
40s	103	33.1
50s	38	12.2
Older than 60	11	3.5
Monthly household income		
Under $2500	91	29.3
$2500 and $4999	105	33.8
$5000 and $7499	35	11.3
$7500 and $9999	22	7.1
Over $10,000	58	18.6
Education level		
Less than college	138	44.4
Bachelor degree	115	37.0
More than a graduate degree	58	18.6
Weekly consumption amount of egg		
Less than 1 egg	40	12.9
2~4 eggs	182	58.5
5~8 eggs	68	21.9
More than 9 eggs	21	6.8

**Table 3:** Results of confirmatory factor analysis.

Construct	Code	Loading	Mean(SD)	AVE	CR
Price fairness	PF1 PF2 PF3 PF4	0.894 0.965 0.923 0.903	2.53 (1.24)	0.849	0.957
Nutritional value	NV1 NV2 NV3 NV4	0.801 0.914 0.735 0.893	4.29 (0.76)	0.703	0.904
Product size	SZ1 SZ2 SZ3 SZ4	0.827 0.921 0.966 0.911	4.17 (0.84)	0.828	0.949
Packaging	PK1 PK2 PK3 PK4	0.904 0.903 0.913 0.889	4.21 (0.83)	0.814	0.946
Hygiene	HY1 HY2 HY3	0.802 0.933 0.906	3.97 (0.88)	0.778	0.912
Trust	TR1 TR2 TR3 TR4	0.794 0.881 0.864 0.911	3.97 (0.89)	0.745	0.921
Loyalty	LY1 LY2 LY3 LY4	0.717 0.849 0.834 0.909	3.92 (0.97)	0.689	0.898

Note: SD stands for standard deviation. Goodness of fit indices: χ^2^ = 589.622, *df* = 303, χ^2^/*df* = 1.946, RMR = 0.047, Normed Fit Index (NFI) = 0.930, Relative Fit Index (RFI) = 0.919, Incremental Fit Index (IFI) = 0.957, Tucker–Lewis Index (TLI) = 0.959, Comparative Fit Index (CFI) = 0.965, Root Mean Square Error of Approximation (RMSEA) = 0.055, CR stands for construct reliability, and AVE is average variance extracted.

**Table 4:** Correlation matrix.

	1	2	3	4	5	6	7
1.Loyalty	0.830						
2.Trust	0.516 *	0.863					
3.Price fairness	0.189 *	0.240 *	0.921				
4. Nutritional value	0.413 *	0.460 *	0.138 *	0.838			
5. Product size	0.488 *	0.517 *	0.292 *	0.396 *	0.907		
6. Packaging	0.467 *	0.574 *	0.129 *	0.385 *	0.673 *	0.902	
7. Hygiene	0.440 *	0.579 *	0.174 *	0.432 *	0.521 *	0.633 *	0.882

Note: * *p* < 0.05, Diagonal is the square root of average variance extracted, SD stands for standard deviation.

**Table 5:** Results of hypothesis testing.

Path	Beta	Critical Ratio	*p*-Value	Results
Price fairness → Trust	0.094	2.813 *	0.005	H1a supported
Price fairness → Loyalty	0.034	0.907	0.190	H1b not supported
Nutritional value → Trust	0.207	3.289 *	0.001	H2a supported
Nutritional value → Loyalty	0.207	2.880 *	0.004	H2b supported
Product size → Trust	0.090	1.267	0.205	H3a not supported
Product size → Loyalty	0.188	2.357 *	0.018	H3b supported
Packaging → Trust	0.256	3.213 *	0.001	H4a supported
Packaging → Loyalty	0.108	1.208	0.227	H4b not supported
Hygiene → Trust	0.257	3.760 *	0.000	H5a supported
Hygiene → Loyalty	0.036	0.468	0.639	H5b not supported
Trust → Loyalty	0.277	3.613 *	0.000	H6 supported

Note: * *p* < 0.05 stands for the critical ratio is significant from the statistical point of view.

## Data Availability

The data presented in this study are available on request from the corresponding author. The data are not publicly available due to privacy.
